# Phthriasis Palpebrarum Mimicking Lid Eczema and Blepharitis

**DOI:** 10.1155/2009/803951

**Published:** 2009-11-30

**Authors:** Burak Turgut, Julide Kurt, Onur Çatak, Tamer Demir

**Affiliations:** Department of Ophthalmology, Fırat University School of Medicine, 23119 Elazig, Turkey

## Abstract

Phthiriasis palpebrarum (PP) is a rare eyelid infestation caused by phthirus pubis. We report a case of PP mimicking lid eczema and blepharitis. A 68-year-old woman had moderate itching in both eyes. Her initial diagnosis was considered to be lid eczema or blepharitis because of findings similar to exfoliative lesions and color changes in eyelids and to excretions over eyelashes. Careful observation revealed many lice and translucent nits, protuberances and hyperpigmentary changes, and the buried lice in both eyelids. No hyperemia or secretion was observed on the lids and in the conjunctiva in both eyes. The patient was treated with pilocarpine hydrochloride 4% drops. At the end of the first week, no louse or nit was present. Although it was known that PP is a rare cause of blepharoconjunctivitis, it might observe as an isolated infestation of the eyelids and this condition can easily be misdiagnosed as lid eczema and blepharitis.

## 1. Introduction

Phthiriasis palpebrarum (PP) is an eyelid infestation caused by phthirus pubis or crab lice. Although pediculus species typically infest the hair, infestation of the cilia and eyelid is rare. Phthiriasis palpebrarum may also be the cause of blepharoconjunctivitis [1, 2]. We report a case of phthiriasis palpebrarum mimicking lid eczema and blepharitis.

## 2. Case Report

**Figure 1:**
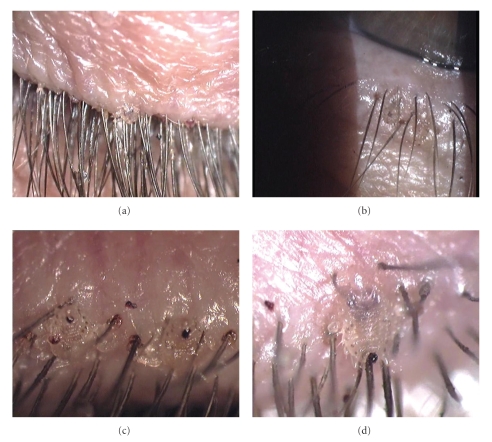
Color photographs showing the lid lesions mimicking excretions over eyelashes (a), color changes mimicking lid eczema on skin of the lower eyelid (b) in the right eye, two lice partially buried into base of eyelashes and clipping eyelashes in the right eye (c), and a louse clipping eyelashes in the left eye (d).

A 68-year-old woman with diabetes mellitus was referred to our outpatient clinic for retinopathy. Her main symptom was itching of her eyelids. On external examination of the periorbital region, the diagnosis was considered to be lid eczema or blepharitis because of the findings similar to exfoliative lesions and color changes in eyelids and excretions over eyelashes (Figures 1(a) and 1(b)). 

**Figure 2:**
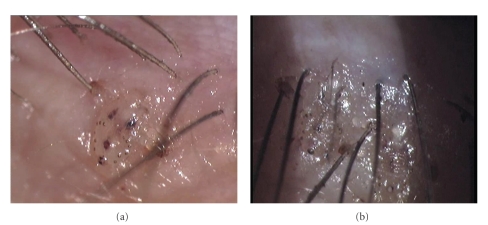
Color photographs showing a louse hardly clipping eyelashes and buried into lid skin (a) and apparently two buried lice into eyelid skin (b) in the right eye.

Her visual acuity was 20/20 in both eyes. Slit-lamp examination revealed two lice on the right eyelashes and one louse around the base of the left one. Additionally, many translucent oval eggs and clipping eyelashes were observed bilaterally (Figures 1(c) and 1(d)). Further observation revealed protuberances and hyperpigmentary changes around the bases of the lashes and buried lice into the lids bilaterally (Figures 2(a) and 2(b)). No hyperemia or secretion was observed over the lids and in the conjunctiva.

**Figure 3:**
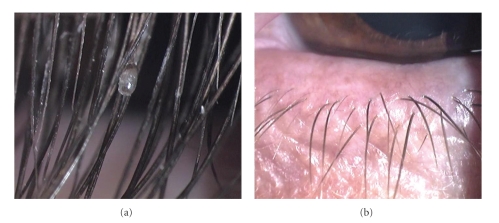
At the second day of the treatment, there is only one nit clipping cilia in the left eye (a) and after the treatment, it is observed that all the lice in eyelids were disappeared (b).

Mechanical removal of the lice and nits was not performed. The patient was treated with pilocarpine hydrochloride 4% applied four times daily. She was consulted for pediculosis capitis in the Dermatology Department and additional treatment was given for the scalp pediculosis. When the patient was reexamined in the next day, it was observed that all the lice disappeared and there was only one nit clipping cilia on the left eyelash (Figure 3(a)). Monotherapy with pilocarpine drops was continued for seven days. On the eighth day, no louse or nit was present (Figure 3(b)). Additionally, the patient and her family were advised to avoid close body contact until the completion of treatment and followup.

## 3. Discussion

Adult lice infest hairs of the scalp, axilla, chest, pubic and rarely, eyebrows, and eyelashes. Infestation of lice on eyebrows or eyelashes is most commonly caused by phthirus pubis, which is transferred by hand contact from the genital area to the eye. Occasionally, isolated palpebral involvement has been described [2–4].

The translucent oval nits which locate into the bases of the eyelashes and on the cilia are often confused with the crusty excretions of seborrheic blepharitis [4]. 

A number of treatment options include mechanical removal with fine forceps, trimming or plucking of eye lashes, traumatic amputation, cryotherapy, argon laser photocoagulation, fluorescein eyedrops 20%, physostigmine 0.25%, lindane 1%, petroleum gel, yellow mercuric oxide ointment 1%, malathion drops 1% or malathion shampoo 1%, and oral ivermectin and pilocarpine gel 4% [1, 4–9].

Although lice are difficult to detect due to its semitransparency and deep burrowing in the lid margin, physicians can observe the parasite's slow movement by careful and prolonged observation. Inspection of the accumulation of translucent oval nits and faeces as the red dish-brown granular material on the base of the lashes may also help diagnosis [1–4].

In our case, all the lice and eggs using pilocarpine 4% drop were destroyed meticulously without any further management. Although the exact mechanism of action of topical pilocarpine is not clearly known, it might be due to its direct cholinergic action causing paralysis of the lice or to its direct pediculicidal action. The patient tolerated the procedure well and no more lice or nit was found on the next visit. 

It is known that PP is a rare cause of blepharoconjunctivitis. Blepharitis with marked conjunctival inflammation, preauricular lymphadenopathy, and secondary infection at the site of lice bite may also be observed [8, 9]. However, we did not observe hyperemia, edema, steamy excretions, or dried excretions on the lids and eyelashes, and conjunctival hyperemia for the diagnosis considering blepharoconjunctivitis. Following careful slit-lamp examination, it was noticed that the findings included the exfoliation, laceration, maceration, and thinning in the lid skin and protuberances due to the buried lice into the base of the eyelashes or follicles. Although we initially conceived as lid eczema and blepharitis, eventually we diagnosed PP without eczema and infection.

 As soon as the diagnosis is made in the cases of PP, to prevent extension of disease, prompt treatment and patient isolation should be considered. The patients with the symptom of pruritus of the eyelids and with clinical findings resembling exfoliation on the surface of lid skin and seborrhea accumulation on eyelashes must carefully be examined by slit lamp in order to avoid misdiagnosis. In the cases diagnosed as having lid eczema and seborrheic blepharitis, lice and nits might easily be overlooked and treatment might remain ineffective. Similar to this presented case, buried lice into the lid skin and into the base of follicle might be overlooked even during biomicroscopic examination.
